# USING MULTICORRESPONDENCE ANALYSES AND CLUSTER ANALYSIS TO CONSTRUCT TYPES OF CARE ARRANGEMENTS

**DOI:** 10.1093/geroni/igac059.703

**Published:** 2022-12-20

**Authors:** Jan Dreyer, Johannes-Michael Bergmann, Kerstin Koehler, Iris Hochgraeber, Christiane Pinkert, Martina Roes, Bernhard Holle

**Affiliations:** German Center for Neurodegenerative Diseases (DZNE), Göttingen, Nordrhein-Westfalen, Germany; German Center for Neurodegenerative Diseases (DZNE), Göttingen, Nordrhein-Westfalen, Germany; German Center For Nuerodegenerative Diseases (DZNE), Witten, Nordrhein-Westfalen, Germany; German Center For Nuerodegenerative Diseases (DZNE), Witten, Nordrhein-Westfalen, Germany; German Center For Nuerodegenerative Diseases (DZNE), Witten, Nordrhein-Westfalen, Germany; German Center for Neurodegenerative Diseases (DZNE), Witten, Nordrhein-Westfalen, Germany; German Center For Neurodegenerative Diseases (DZNE), Witten, Nordrhein-Westfalen, Germany

## Abstract

Most persons with dementia live at home and are cared for by family carers and professional carers. Together, they form care arrangements to address the needs of persons with dementia. The aim of this study is to (1) uncover the underlying structures of home-based care arrangements for persons living with dementia, (2) construct types of these care arrangements, and (3) compare these types with regard to their stability. In this secondary analysis, data from 320 care arrangements for persons with dementia are analysed using multiple correspondence analysis and hierarchical cluster analysis. The multiple correspondence analysis identified 27 axis that explained the entire variance between all care arrangements. The subsequent cluster analysis identified four types of care arrangements. Two types included spouse-centred care arrangements, and two types included child-centred care arrangements at different phases of the dementia and care trajectory. The types differ with regard to their stability.

